# The impact of oxidative stress-induced mitochondrial dysfunction on diabetic microvascular complications

**DOI:** 10.3389/fendo.2023.1112363

**Published:** 2023-02-07

**Authors:** Ziwei Zhang, Qingxia Huang, Daqing Zhao, Fengmei Lian, Xiangyan Li, Wenxiu Qi

**Affiliations:** ^1^ College of Traditional Chinese Medicine, Changchun University of Chinese Medicine, Changchun, China; ^2^ Key Laboratory of Active Substances and Biological Mechanisms of Ginseng Efficacy, Jilin Provincial Key Laboratory of Biomacromolecules of Chinese Medicine, Ministry of Education, Northeast Asia Research Institute of Traditional Chinese Medicine, Changchun University of Chinese Medicine, Changchun, China; ^3^ Research Center of Traditional Chinese Medicine, The Affiliated Hospital to Changchun University of Chinese Medicine, Changchun, Jilin, China; ^4^ Guang’anmen Hospital, China Academy of Chinese Medical Sciences, Beijing, China

**Keywords:** diabetes mellitus, diabetic microvascular complications, mitochondria, oxidative stress, oxidative phosphorylation, mitochondrial DNA

## Abstract

Diabetes mellitus (DM) is a metabolic disease characterized by chronic hyperglycaemia, with absolute insulin deficiency or insulin resistance as the main cause, and causes damage to various target organs including the heart, kidney and neurovascular. In terms of the pathological and physiological mechanisms of DM, oxidative stress is one of the main mechanisms leading to DM and is an important link between DM and its complications. Oxidative stress is a pathological phenomenon resulting from an imbalance between the production of free radicals and the scavenging of antioxidant systems. The main site of reactive oxygen species (ROS) production is the mitochondria, which are also the main organelles damaged. In a chronic high glucose environment, impaired electron transport chain within the mitochondria leads to the production of ROS, prompts increased proton leakage and altered mitochondrial membrane potential (MMP), which in turn releases cytochrome c (cyt-c), leading to apoptosis. This subsequently leads to a vicious cycle of impaired clearance by the body’s antioxidant system, impaired transcription and protein synthesis of mitochondrial DNA (mtDNA), which is responsible for encoding mitochondrial proteins, and impaired DNA repair systems, contributing to mitochondrial dysfunction. This paper reviews the dysfunction of mitochondria in the environment of high glucose induced oxidative stress in the DM model, and looks forward to providing a new treatment plan for oxidative stress based on mitochondrial dysfunction.

## 1 Introduction

DM is characterized by chronic and persistent hyperglycemia and causes macrovascular and microvascular complications, which is a major cause of end-stage renal disease, blindness, and amputation today (1–4). As of 2014, there were approximately 387 million people with DM worldwide (8.3% of the world’s population) and the number of people with DM is expected to increase to 640 million by 2040 (5, 6). The cost of diabetic microvascular complications is a major component of overall treatment costs and places a heavy burden on society and families (7).

The development and progression of DM has complex pathophysiological mechanisms, with inflammation, autophagy dysregulation, oxidative stress, and hemodynamic dysregulation all involved in the progression of the disease (8–11). In this regard, oxidative stress is an important part of disease development, and chronic hyperglycaemia promotes an imbalance between the production of free radicals and the scavenging capacity of the body’s antioxidant system. Mitochondria play an important role in the process of oxidative stress in cells. Mitochondria are the main site of cellular respiration in the body and the central organelle for the production of adenosine triphosphate (ATP) (12). Continuous high glucose stimulation leads to impaired mitochondrial electron transport and promotes the production of ROS, which in turn causes damage to the mitochondria themselves and mitochondrial DNA (mtDNA), leading to impaired ATP synthesis, apoptosis, and activation of downstream inflammatory and fibrotic signaling pathways, contributing to disease progression (13–15). Scientists are now proposing that improving the antioxidant capacity of cells may be an important strategy for treating DM and complications, with some experiments in animals and humans and some results, but the evidence-based clinical support for anti-oxidative stress therapies is still insufficient (16–18). Based on this, the aim of this review is to explore together the important role of mitochondria in the process of oxidative stress in DM through abnormal mitochondrial oxidative phosphorylation (OXPHOS) and mtDNA damage, and to outline the efforts made by present-day scientists towards repairing mitochondrial function in the hope of providing new ideas for future scientific experiments.

## 2 Oxidative stress and ROS

The concept of oxidative stress was first introduced by the German scientist Helmut Sies and refered to the imbalance of oxidants and antioxidants that can cause damage to the organism (19). The concept of “oxidative stress” was later extended to refer to a pathological phenomenon resulting from an imbalance between the production of free radicals and the scavenging function of the antioxidant system, and is closely linked to the development of diseases such as chronic obstructive pulmonary disease, Alzheimer’s disease, cancer, DM, hypertension and age-related diseases (20–26).

**Figure 1:**
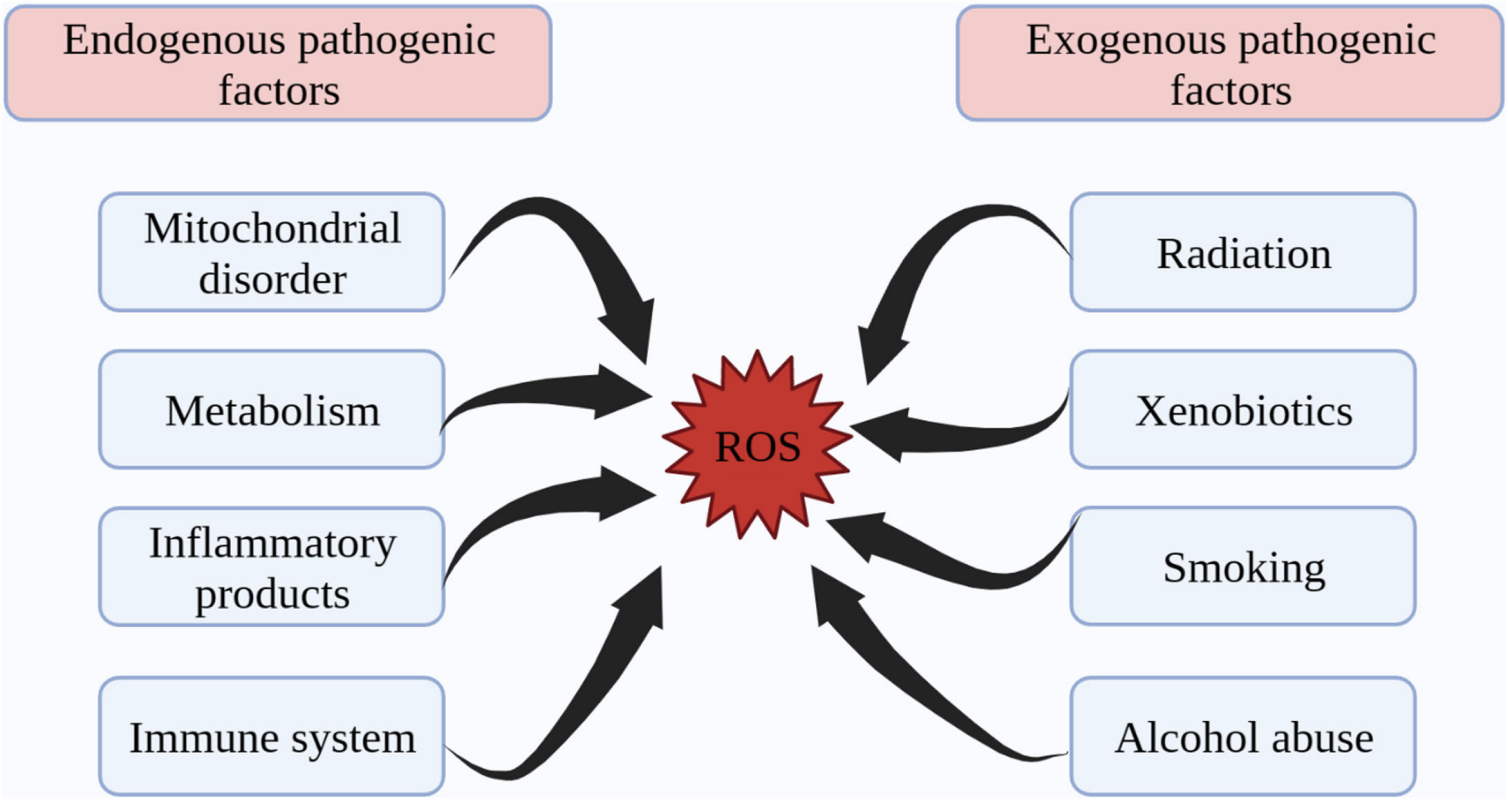
Endogenous and exogenous factors in ROS production. ROS generation is divided into two aspects: endogenous pathogenic factors and exogenous pathogenic factors. Endogenous pathogenic factors include mitochondrial disorder, metabolism, inflammatory products, immune system, etc. Exogenous pathogenic factors include radiation, xenobiotics, smoking, alcohol abuse, etc. ROS, reactive oxygen species.

Structurally, free radicals are highly reactive substances containing at least one unpaired electron and are active derivatives of ROS and reactive nitrogen species (RNS), which are closely associated with the initiation of oxidative stress (27, 28). Both endogenous and exogenous stimulation lead to pathological increases in ROS and promote oxidative stress. Endogenous factors such as metabolic factors, mitochondrial damage, immune system dysregulation, and inflammatory products induce the production of ROS (29–32). Exogenous ionizing radiation, xenobiotics, ultraviolet light, alcohol abuse and smoking contribute to the onset and progression of aging and metabolic disease by promoting ROS production in the body (33–38) (**Figure 1**).

The intracellular ROS is mainly composed of O_2_^.-^ [reduced from O_2_ by electrons through the electron transfer chain (ETC)] and its derivatives. The three main reactive substances of ROS are superoxide anion (O_2_^.-^), hydroxyl radical (•OH) and hydrogen peroxide (H_2_O_2_), with •OH being the most reactive (39, 40). Superoxide dismutase (SOD), the first line of defense of the cellular antioxidant defense system, promotes the production of the superoxide O_2_^.-^intermediating H_2_O_2_: 2O_2_^.-^+2H^+^→H_2_O_2_+O_2_ (41). Subsequently reduced by catalase (CAT) to non-toxic H_2_O: 2H_2_O_2_→2H_2_O+O_2_; or catalyzed by glutathione peroxidase (GSH-Px) to produce H_2_O: 2GSH+H_2_O_2_→2H_2_O+GSSG, all of which complete the antioxidant scavenging process and constitute the antioxidant enzymatic response system of the organism, maintaining the intracellular redox balance (41, 42). Fe^2+^ and Cu^+^ are capable of redox reactions with unpaired electrons, destroying the structure and function of proteins, nucleic acids and lipids and cross-linking with these macromolecules to produce toxic substances (19). Activation of mitochondrial permeability transition pore (MPTP) promotes the release of ROS (43). Also, the vicious cycle of oxidative stress is facilitated by an increase in toxic substances such as malondialdehyde (MDA), and 4-hydroxy-2-nonenal (4-HNE), which are lipid peroxidation products (44, 45). Likewise, advanced glycosylation end products (AGEs) further promote ROS production, induce overexpression of endothelial angiopoietin (Ang)-2, promote cellular sensitivity to pro-inflammatory factors such as vascular cell-adhesion molecule (VCAM)-1, and contribute to the progression of diabetes-related vascular disease (46). AGEs are non-enzymatic glycosylated forms of free amino acids that result from the interaction of glucose with lipids or proteins, bind to receptors to promote inflammation and oxidative stress, and are important in contributing to glomerulosclerosis and mesangial hypertrophy in DKD (47–49). On the other hand, ROS acts as an agonist of NF-κB signaling pathway to initiate the activation of downstream inflammatory signaling pathways and promotes the release of inflammatory factors such as IL-1β, TNF-α, intercellular adhesion molecule-1 (ICAM-1) and monocyte chemotactic protein-1 (MCP-1) (50–54).

**Figure 2:**
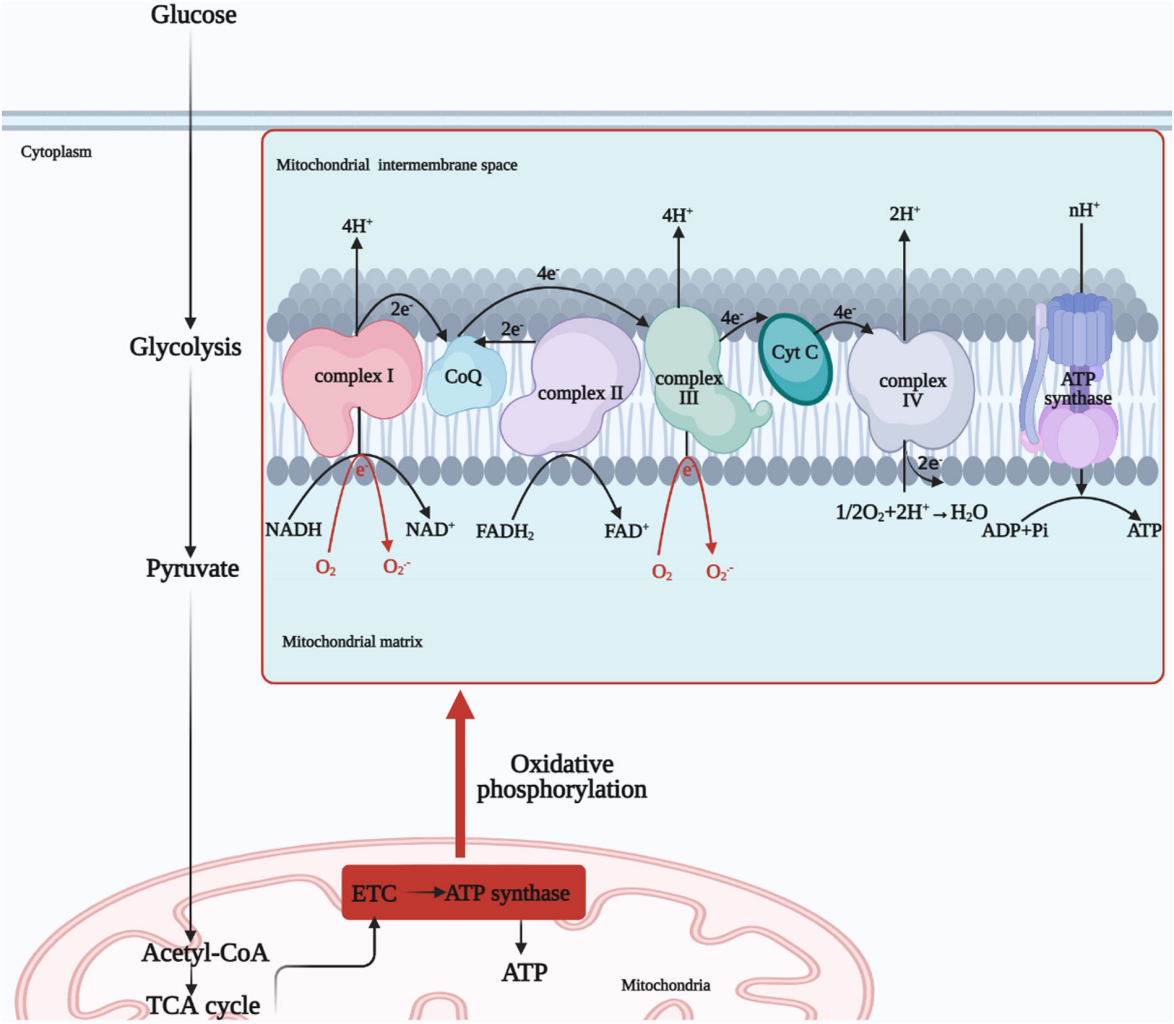
Mitochondrial OXPHOS process. Glucose undergoes glycolysis to produce pyruvate, which is oxidized to acetyl-coenzyme A or carboxylated to produce oxaloacetate, which enters the mitochondrial matrix for the TCA cycle. And finally NADH and FADH_2_, electron donors, undergo electron transfer by the ETC to produce H_2_O from O_2_ and ATP by the action of ATP synthase, completing OXPHOS process. NADH, nicotinamide adenine dinucleotide; FADH_2_, flavin adenine dinucleotide; ETC, electron transfer chain; TCA, tricarboxylic acid; ATP, adenosine triphosphate, ADP, adenosine diphosphate; OXPHOS, oxidative phosphorylation; Cyt C, cytochrome c; O_2_^.-^, superoxide anion.

We know that mitochondria are the main site of ROS production and a target organelle for oxidative stress (55). The ETC is the central component of the mitochondria for functional operation, and the sequential transfer of electrons through the ETC to complex IV creates an electrochemical proton gradient that drives the F_1_F_0_ ATP synthase to produce ATP for OXPHOS (56). In addition, mitochondrial ROS production is increased and morphology is altered in high blood glucose environment of DM. Further, prolonged high glucose stimulation results in more electron donors such as NADH and FADH_2_ being produced in the tricarboxylic acid (TCA) cycle, and too many electron donors entering the ETC, leading to a maximum mitochondrial voltage gradient (57). Uncoupling proteins (UCPs) reduce ROS production by dissipating the proton motive force and increasing the rate of electron transfer in the ETC (58). At the same time, molecular oxygen reacts prematurely to produce superoxide O_2_^.-^, which exceeds the antioxidant scavenging capacity and leads to oxidative stress, causing impairment of cellular respiratory function, promoting apoptosis and ultimately leading to dysfunction of diseased tissues and systems (59, 60) (**Figure 2**). It is reported that NADH oxidoreductase (complex I) and cytochrome bc_1_ oxidoreductase (complex III) are the main sites for the production of ETC superoxide (61). In addition to this, much of the literature has further linked mitochondrial OXPHOS function and mtDNA damage closely to oxidative stress (55, 62). High glucose-induced oxidative stress and mitochondrial dysfunction interact to promote the progression of DM and its complications (63). One article focused on the relationship between diabetic renal tubular injury and mitochondrial dysfunction, contributing to increased ROS production and metabolic abnormalities such as abnormal mitochondrial autophagy, and the article suggested that mitochondrial dysfunction may contribute to early diabetic tubulopathy (64). In addition to mitochondria, cytoplasmic NADPH oxidase (Nox) is the main source of cytoplasmic ROS (55). Further, cytoplasmic ROS could increase mitochondrial ROS production by continuously damaging mitochondria (6). Overall, mitochondria remain the main source of endogenous ROS (43). Therefore, we will next focus on the relationship between mitochondria and oxidative stress in the next sub-section.

It is important to note that the physiological level of ROS plays an important role in signaling, defense against infection and maintenance of redox homeostasis, only excessive ROS production leads to adverse effects of oxidative stress (65–67).

## 3 Physiological functions of mitochondria and oxidative damage

### 3.1 OXPHOS

In mitochondria, OXPHOS reactions use over 95% of O_2_ to produce ATP, and a small amount of ROS are produced daily as OXPHOS by-products (68). However, when the production of ROS exceeds the scavenging capacity of the antioxidant defense system, it can lead to oxidative stress.

Glucose is one of the main sources of energy for the body and is glycolysed in the cytoplasm to produce pyruvate, which is oxidized to acetyl-coenzyme A or carboxylated to produce oxaloacetate, which enters the mitochondrial matrix for the TCA cycle (69). Subsequent production of nicotinamide adenine dinucleotide (NADH) and flavin adenine dinucleotide (FADH_2_) provides the respiratory substrate for the ensuing OXPHOS process, which drives ATP production (70).

Mitochondria have a bilayer membrane structure, with the electron transport chain localized to the inner mitochondrial membrane (IMM), which is inlaid with four protein complexes, namely NADH oxidoreductase (complex I) (the largest subunits enzyme complex in the ETC), succinate dehydrogenase (complex II), cytochrome bc_1_ oxidoreductase (complex III) and cytochrome c oxidase (complex IV) (71). There are also two free-moving electron transport carriers on the ETC, the lipid-soluble Q and the water-soluble cyt-c, and these six components together form the ETC supercomplex (72).

Two electrons from the TCA metabolite NADH in complex I are passed to Q and reduced to QH_2_ (73). At the same time, the Fe-S cluster conformational change induces proton translocation and pumps four protons into the mitochondria intermembrane space (IMS) (74). Complex II is also an important carrier for the transfer of electrons, with FADH_2_ transferring electrons to Q *via* the Fe-S cluster. Protons are required for the reduction of Q, so there is no net proton increase in the IMS (75). Complex III transfers electrons of QH_2_ to cyt-c, cyt-c that gets electrons will be reduced, and the completion of the Q-cycle requires the pumping of four protons into the IMS (75). The reduced cytochrome carries electrons into complex IV, where they are eventually passed to the binuclear center of complex IV to complete the reduction of O_2_, also known as one molecule of O_2_ to produce two molecules of H_2_O (76). Eight protons in the matrix are consumed in this process, four of which are pumped into the IMS (77). At this point, the high concentration of protons in the IMS constitutes the electrochemical gradient responsible for the energy storage of the mitochondria (78). F_1_F_0_ ATP synthase, also known as complex V, transfers protons from the IMS to the matrix and controls the threshold of the MMP, at which point ATP synthase undergoes structural changes that promote ADP phosphorylation to produce ATP (79, 80). In addition, UCPs lower the membrane potential by transferring protons from the IMS to the matrix and uncouple from the ATP synthesis process, forming a switch for ATP synthesis with ATP synthase (81) (**Figure 2**).

### 3.2 Structure and function of mtDNA

mtDNA is a double-stranded circular structure (consisting of light and heavy strands), localizes in the mitochondrial matrix, closing to IMM (82). The human mtDNA is 16,569 bp in length and consists of 37 genes, 22 tRNAs, 2 rRNAs and a non-coding region displacement-loop (D-loop) (83, 84). However, the non-coding region controls the transcription and translation of mitochondrial proteins, but the high sequence mutagenicity in this region makes the mtDNA mutation rate approximately 10-20 times higher than nuclear DNA, with relevance to diseases such as aging and cancer (85–87). Moreover, due to the tight arrangement of genes in the ring structure of mtDNA, some genes overlap, and lack of histone protection, it is vulnerable to ROS generated by oxidative stress process, resulting in persistent damage to mtDNA (88, 89).

Mitochondria have a different DNA genetic system from nuclear DNA. mtDNA is responsible for encoding some of the mitochondrial proteins (such as the protein complexes that make up the ETC) and involved in mitochondrial biogenesis and signaling, with semi-autonomous genetic characteristics (82, 90, 91). Therefore, mtDNA is important for OXPHOS. Mutations in mtDNA and epigenetic changes can lead to blocked electron transport in the ETC, reducing ATP synthesis and promoting apoptosis.

Further, transcription and packaging factor (TFAM), and transcription elongation factor (TEFM), essential cofactors for mtDNA replication and transcription, play an important role in the assembly and distribution of mtDNA-protein complexes and are thought to alleviate insulin resistance-induced oxidative stress (90, 92–94).

**Figure 3:**
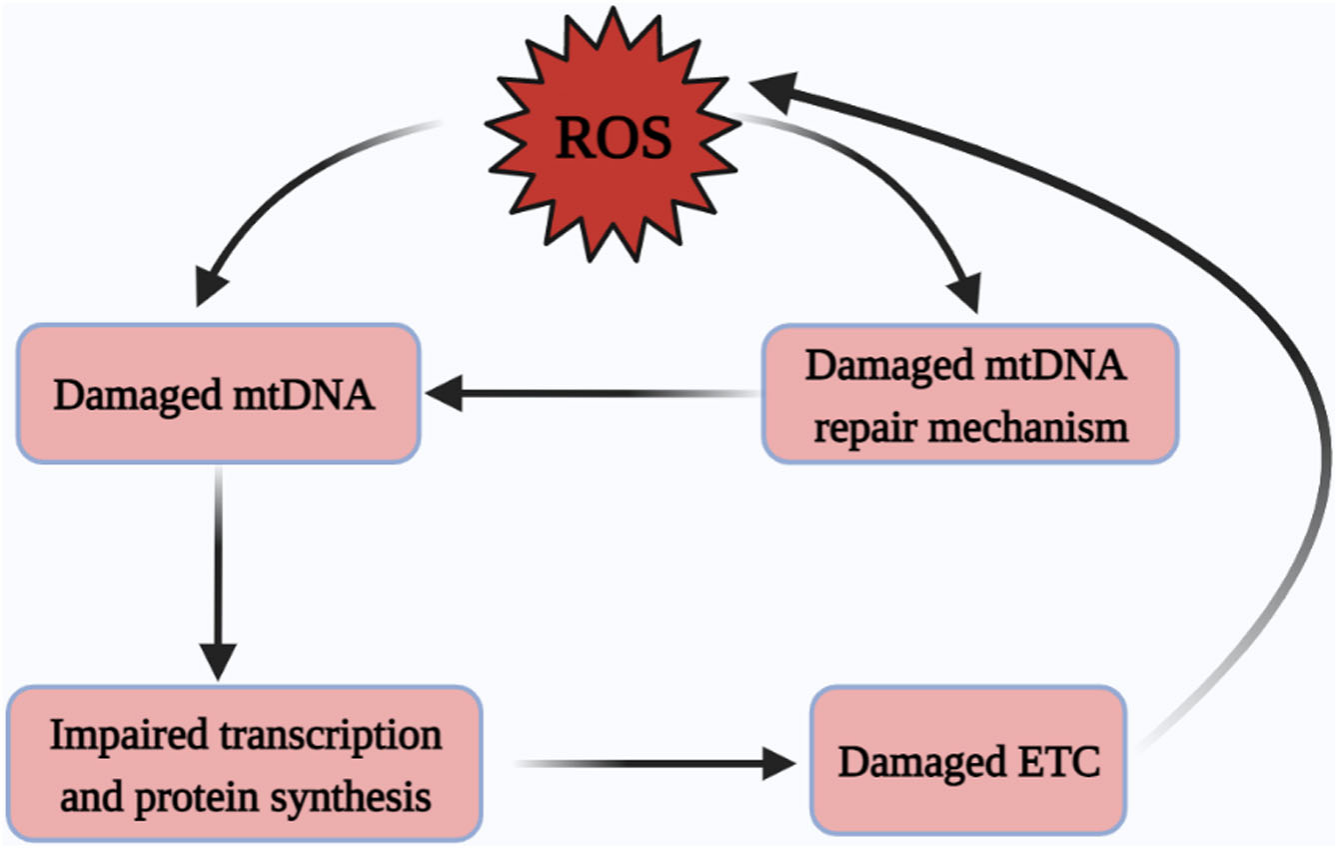
Excessive ROS damages mtDNA. ROS drives damage to mtDNA and the mtDNA repair systems, yet the damage of mtDNA repair systems is also an important contributor to mtDNA damage, followed by damage to mitochondrial protein-related transcription and synthesis pathways, ultimately leading to a vicious cycle of ETC abnormalities and subsequent the excessive generation of ROS. ROS, reactive oxygen species; mtDNA, mitochondrial DNA; ETC, electron transfer chain.

In addition, the organism equips mtDNA with DNA repair systems, and base excision repair (BER) is considered to be the main repair mechanism (95). BER maintains the normal structure of mtDNA by eliminating base mismatches caused by methylation, oxidation and alkylation, and by cleaving, gap-filling and connecting the structure of mtDNA (96). In addition, mismatch repair (MMR), homologous recombination (HR) and non-homologous end joining (NHEJ) are also important repair pathways of mtDNA (97). It has been documented that base mismatches caused by elevated ROS can be recognized and excised by the BER pathway to maintain the functional and structural integrity of mtDNA (98–100). Similarly, *in vitro* and *in vivo* studies of the MMR pathway in high glucose environments have shown that high glucose environments induce damage to mtDNA and also impair the repair of the MMR pathway (101) (**Figure 3**).

## 4 The relationship between oxidative stress-induced mitochondrial damage and diabetic microvascular complications

Oxidative stress, an important trigger for the development of DM and its complications, is characterized by excessive ROS production and intracellular oxidative damage. Oxidative stress can lead to alterations in mitochondrial morphology and function, inducing structural changes and functional abnormalities in macromolecules such as proteins, lipids and nucleic acids, ultimately leading to apoptosis and accelerating the progression of diabetic microvascular complications such as diabetic retinopathy (22, 102–105). In the following we will focus on mitochondria as an entry point to explore the impact of oxidative stress-induced mitochondrial dysfunction on diabetic microvascular complications.

### 4.1 Impaired OXPHOS and recovery in diabetic microvascular complications

OXPHOS occurs in mitochondria and is the main process involved in supplying energy for cellular respiration and ATP synthesis (106). ETC is the central element of the OXPHOS process, with the sequence of complexes (I-IV) working to achieve electron and proton transfer, creating MMP to store energy for the next work of ATP synthase (75). However, prolonged hyperglycaemic stimulation leads to abnormal electron transfer, resulting in increased production of ROS such as superoxide, inducing the onset of oxidative stress (107). Therefore, excessive ROS is one of the main factors leading to impaired OXPHOS function.

#### 4.1.1 Diabetes kidney disease (DKD)

In high glucose induced podocytes, the superoxide levels were found to be increased while MMP expression and mitochondrial number were found to be decreased. However, overexpression of SIRT6 was shown to reverse this phenomenon (108). Dioscin effectively reduced blood glucose and markers of renal impairment in diabetic rats, reversed mitochondrial respiratory chain disorders, increased the activity of SOD and CAT antioxidant enzymes and reduced the level of ROS (109). Jujuboside A modulated mitochondrial respiratory chain complex protein expression in T2DM rats, improved respiratory chain function, reduced ROS levels, increased SOD, CAT and GPX expression, and downregulated apoptotic protein expression (110). In a study evaluating Abroma augusta L. (Malvaceae) leaf extract on T2DM-related nephropathy and cardiomyopathy in experimental rats, it was observed that redox homeostasis was disrupted, intracellular NAD and ATP levels were reduced and mitochondria-dependent apoptotic pathways were activated in T2DM state (111). Studies have shown that soluble klotho protein (referred to as rKL, known as an inhibitor of aging) reduced albuminuria, restored mitochondrial function and reduced ROS production in db/db mice. Moreover, in high glucose-induced mouse proximal tubular cells, rKL treatment alleviated OXPHOS impairment and induced mitochondrial repair *via* the PGC-1α-AMPK pathway (112). To investigate the effects of a high-fat diet on oxidative stress in wild-type and RAGE (receptors for AGEs) deficient mice, it was shown that RAGE can regulate mitochondrial respiratory chain function and oxidative stress in flounder muscle (113). Metformin, a classical hypoglycemic agent, promoted normalization of energy status and biochemical alterations, elevated ATP and lowered AMP, inhibited TNF-α and IL-6 pro-inflammatory gene expression, and exerted protective function of kidney in DKD rats (114). C3a induced mitochondrial fragmentation in podocytes, promoted mitochondrial depolarization, decreased SOD expression and increased ROS production, contributing to abnormal cellular energy metabolism, but this phenomenon could be inhibited by SS-31 (115). Knockdown of heat shock protein 60 (HSP60) in high-glucose-induced canine renal tubular cells showed that HSP60 regulated protein aggregation and ATP production in renal tubular cells (116). In high glucose and angiotensin II (ANG II)-induced HK-2 cells, increased p66Shc (promoter of apoptosis) and p-p66Shc were accompanied by increased ROS. The researchers made in-depth research and concluded that p66Shc mediated high glucose and ANG II-induced mitochondrial dysfunction *via* protein kinase C (PKC)-Β and peptidyl-prolyl isomerase (Pin1) pathways, decreased MMP, promoted cyt-c leakage and increased the apoptotic protein caspase-9 (117). In another study, one of the mechanisms by which p66Shc promoted DKD was that p66Shc promoted disruption of mitochondrial dynamics, enhanced Mfn1-Bak interactions leading to loss of mitochondrial voltage potential, cyt-c release, excessive ROS production and apoptosis (118). In contrast, coagulation protease activated protein C and normalized MMP through epigenetic inhibition of p66Shc (119); also Obacunone exhibited nephroprotective effects that inhibited oxidative stress and mitochondrial dysfunction (120). Purple Rice Husk improved mitochondrial function through the PGC-1α/SIRT3/SOD2 signaling pathway and reduced oxidative damage in renal tissue (121). Knockdown of the mitochondrial uncoupling protein UCP-2 increased uncoupling through adenine nucleotide transport proteins and reduced oxidative stress in the diabetic kidney in rat models (122). In STZ-induced diabetic mice, phillyrin reduced blood glucose and serum creatinine levels, increased Bcl-2/Bax ratio, reduced cyt-c leakage into the cytoplasm and inhibited apoptosis through the PI3K/Akt/GSK-3β signaling pathway (123). Genistein protected podocytes integrity, increased MMP, improved mitochondrial function and inflammatory status in rats with diabetic nephropathy by inhibiting the MAPK/NF-κB pathway (124). Telmisartan increased the MMP of glomerular endothelial cells induced by high glucose, and reduced the levels of 8-hydroxy-2-deoxyguanosine (8-OHdG) and MDA to alleviate oxidative stress (125). The complex I inhibitor rotenone (ROT) reduced ROS production and increased MMP and PGC-1α-controlled mitochondrial biogenesis in STZ and different inflammatory factor-induced mouse pancreatic β-cell line Min6 cells, suggesting that inhibition of complex I might be an effective strategy to protect β-cells in T1DM (126). Another study had shown that ROT could also correct over-activated biological processes, increasing the ratio of reduced glutathione (GSH) and nicotinamide adenine dinucleotide phosphate (NADPH) to its oxidized form, leading to redox balance (127). Resveratrol alleviated proteinuria, reduced ROS and MDA levels, restored SIRT1 and PGC-1α expression in kidney tissue. Resveratrol inhibited mitochondrial oxidative stress *via* SIRT1/PGC-1α, improved podocytes respiratory chain complex I and III activity, increased MMP and inhibited cyt-c release from mitochondria to the cytoplasm (128). In palmitic acid (PA) and oleic acid induced podocytes, PA was found to induce mitochondrial superoxide and H_2_O_2_ production (129). To investigate the role of SIRT3 deficiency on mitochondrial damage, researchers fed SIRT3-deficient mice a high-fat diet, resulting in mitochondrial dysfunction (involving abnormalities in OXPHOS, MMP and energy metabolism) and ultrastructural changes (130). In addition, a number of studies had also shown a close pathological link between impaired OXPHOS process and DKD (131–134).

The selective SIRT1 agonist BF175 was shown to prevent high glucose-induced mitochondrial damage and reduce superoxide production (135). Salvianolate effectively inhibited the generation of superoxide derived from NOX4 (mainly located in IMM) and reduced podocyte apoptosis (136). In the STZ-induced DKD rat model, the researchers observed a significant increase in blood creatinine and urine protein, as well as in ROS and MDA levels in the model group compared to the control group (137). In H_2_O_2_-induced HBZY-1 cells, Nepeta angustifolia inhibited H_2_O_2_ by increasing MMP, reducing ROS and MDA levels while inhibiting apoptosis (138). It had been shown that in addition to elevated biochemical parameters associated with kidney damage, STZ-induced diabetic rats also increased ROS production, reduced antioxidant defenses *in vivo*, and ultimately initiated mitochondria-dependent apoptosis (139). Increased ROS formation, elevated lipid peroxidation products and oxidative DNA damage, and mitochondrial apoptosis were observed in kidney tissue in STZ-induced diabetic mice, but dietary eicosapentaenoic acid inhibited this phenomenon by modulating hypoxia-inducible factor (HIF)-1α (140). Another study showed that Erythropoietin alleviated mitochondrial dysfunction, inhibited mitochondrial fragmentation and ROS production, and promoted autophagic flux *in vitro* (141). CAT deficiency increased ROS production and fibronectin expression in DKD mice and murine mesangial cells, demonstrating that endogenous catalase played an important role in the maintenance of mitochondrial function and protected the kidney from oxidative stress (142). Ferulic acid inhibited ROS production and apoptosis and induced autophagy in STZ-induced diabetic rats (143). In addition, other researchers found that Nox4 knockdown reduced NADPH oxidase activity, accompanied by reduction in high-glucose-induced superoxide, yet mitochondrial Nox4 expression was increased in the renal cortex of diabetic rats, demonstrating the role for Nox4 in the regulation of mitochondrial function (144). Adropin improved lipid metabolism and renal function in diabetic mice, regulated blood glucose and lipids, inhibited ROS production, improved lipid deposition and down-regulated lipoprotein expression (145). G Protein-Coupled Bile Acid Receptor TGR5 improved indicators of renal injury in db/db mice, upregulated regulators of mitochondrial biogenesis, reduced lipid accumulation and H_2_O_2_ production and increased SOD2 activity; similarly, similar results were observed in high glucose-induced podocytes (146).

#### 4.1.2 Diabetic retinopathy (DR)

In a high glucose-induced retinal ganglion cells (RGC) model, Hesperidin (Citrus Flavonone) restored mitochondrial function, prevented loss of MMP and cyt-c release into the cytoplasm, prevented ROS production, increased intracellular levels of antioxidant enzymes and inhibited apoptosis (147). A study on metabolic memory of mitochondrial oxidative damage found that in primary rat retinal endothelial cells (rRECs) cultured in high glucose, MMP and cyt-c levels decreased and ROS levels increased in the model group compared to the control group as the duration of high glucose culture increased, suggesting that metabolic memory of mitochondrial oxidative damage can lead to DR (148). Another study on Berberine (BBR) showed that BBR alleviated oxidative stress (inhibited cyt-c leakage and ROS production and increased antioxidant enzyme levels) in diabetic rats and high glucose-induced Müller cells by inhibiting the NF-κB signaling pathway, thereby preventing DR (149). Leakage of cyt-c and increased accumulation of Bax in mitochondria in STZ-induced diabetic rats and high glucose cultured retinal endothelial cells and pericytes, which were inhibited by SOD and its mimics (150). In another study, in addition to demonstrating the protective effect of manganese superoxide dismutase (MnSOD) on the retina, it was also demonstrated that complex III might be a more significant source of superoxide compared to complex I (151). It had been suggested that MTP-131 (a novel mitochondrial targeting peptide) alleviated H_2_O_2_-induced oxidative stress in RGC-5 (blocking MMP depolarization and cyt-c release, reducing ROS production and preventing apoptosis) (152). NaHS (donor of H_2_S) blocked retinal abnormalities in diabetic rats and alleviated DR by inhibiting mitochondrial dysfunction and NF-κB activation (153).

In high glucose-induced and platelet-derived growth factor-induced retinal pigment epithelial cells, researchers found that SIRT3 knockdown led to epithelial-mesenchymal transition and migration of epithelial cells, which was alleviated by overexpression of SIRT3. Further studies revealed that the cause was knockdown of SIRT3 leading to overproduction of mitochondrial ROS, suggesting the role for SIRT3 in inhibiting mitochondrial oxidative stress (154). In a vitro study, 670 nm photobiomodulation reduced high glucose-induced ROS production and maintained mitochondrial integrity in rat Müller glial cells (155). In an STZ-induced mouse experiment, STZ induced an increase in cytoplasmic and mitochondrial ROS, accompanied by lipid peroxidation and apoptosis, and a decrease in GSH and GSH-Px as well as optic nerve activity and vitamin A levels, which could be reversed by selenium and resveratrol (156). Some scientists investigated the mechanism of oxidative stress induced by high glucose in RGC-5, and concluded that high glucose induced ROS production, disrupted mitochondrial mechanisms (MMP, mtDNA and mitochondrial mass damage) and antioxidant mechanisms, and triggered the production of downstream inflammatory factors and neurodegenerative markers (157). A study on green tea (Camellia sinensis) and antioxidant vitamins showed that green tea and vitamins reduced retinal superoxide production and that green tea improved inhibition of ETC and complex III activity, but promoted tissue collagen matrix glyco-oxidation (150).

#### 4.1.3 Diabetic peripheral neuropathy (DPN)

Phosphocreatine (PCr, a high-energy phosphate compound) prevented oxidative stress and promoted normalization of mitochondrial function *in vivo* and vitro experiments: PCr acted on complex I and complex II of the mitochondrial respiratory chain to increase cellular respiration and reduce ROS, and might be a potential drug for the treatment of diabetes-related neurodegenerative diseases (158). Salvianolic Acid A (SalA) inhibited high glucose-induced mitochondrial damage in Schwann RSC96 cells by modulating nuclear factor erythroid 2-related factor 2 (Nrf2): SalA scavenged mitochondrial ROS, reduced MMP, increased ATP production and upregulated OXPHOS-related gene expression; and alleviated abnormal glucolipid metabolism in KK-Ay mice, exerting peripheral neuroprotective effects (159). In contrast, high glucose induction led to abnormal changes on mitochondrial superoxide, MMP and neurosynaptic growth in Neuro2a cells, STZ-induced abnormalities in motor/nerve conduction and neuroblood supply in diabetic rats, and polydatin improved mitochondrial dysfunction and biogenesis *via* SIRT1/Nrf2 (160). Long chain fatty acids induced mitochondrial dysfunction of Schwann cells, while overexpression of long chain acyl CoA synthetase 1 improved mitochondrial coupling efficiency, reduced proton leakage, and improved mitochondrial function (161). Human neuroblastoma SH-SY5Y cells exposed to high glucose levels reduced neuropil numbers, downregulated uncoupling protein (UCP) 3, increased MMP and ROS levels, while insulin-like growth factor type 1 normalized these changes (162).

An *in vitro* study of quercetin showed that quercetin reduced high glucose-induced ROS production in RSC96 cells and improved mitochondrial morphological abnormalities and DNA damage, as well as peripheral nerve hypofunction in lesioned mice (163). In high glucose-induced Schwann cells, puerarin inhibited ROS production and mitochondrial depolarization and prevented apoptosis (164). Additionally, it had also been shown that Fuzi protected Schwann cells induced by high glucose, prevented excessive ROS production and apoptosis, and had neuroprotective effects (165).

### 4.2 mtDNA damage and recovery in diabetic microvascular complications

#### 4.2.1 DKD

Increased urinary 8-OHdG was detected in DKD-sensitive DBA/2J mice and human DKD specimens and showed a correlation between glomerular endothelin-1 receptor type A expression and increased mtDNA damage (166). The combination of dietary fenugreek (Trigonella foenum-graecum) seeds and onion (Allium cepa) was effective in reducing STZ-induced oxidative stress, lowering triglyceride and total cholesterol levels, reducing 8-OHdG and DNA fragmentation, and eliminating mtDNA deletions (167). In addition, salidroside alleviated renal fibrosis and kidney damage in DKD mice, and promoted the increase of mtDNA copy number and mitochondrial biogenesis (168).

#### 4.2.2 DR

In high glucose-induced retinal endothelial cells, researchers found increased damage to mtDNA and DNA repair mechanisms and decreased expression of genes responsible for encoding the ETC protein complex, however, overexpression of MnTBAP or MnSOD suppressed this phenomenon (169). Similarly, another study also pointed out that high glucose-induced mtDNA damage led to excessive ROS production and further promoted mtDNA damage, leading to a vicious cycle of oxidative stress (170). Hydrogen sulfide is an endogenous gastransmitter signaling molecule with antioxidant properties, and its donor GYY4137 exhibited antioxidant effects in STZ-induced diabetic mice by resisting mtDNA damage, promoting Cytb transcription, limiting ROS production and inhibiting increased mitochondrial membrane permeability (171). An interesting study found that mtDNA and its repair/replication mechanism were significantly associated with the course of DM: early mtDNA repair/replication enzymes increased compensatorily, and as the disease progressed the repair/replication mechanism was disrupted and the mtDNA copy number decreased significantly (172).

#### 4.2.3 DPN

A study comparing differences in mtDNA and transcript levels between diabetic and PGC-1α^(-/-)^ diabetic mice found that PGC-1α^(-/-)^ exacerbated neurological abnormalities in diseased mice, promoted mtDNA damage and protein oxidation, and led to more severe mitochondrial degeneration, demonstrating that modulation of PGC-1α may be a strategy for treating DPN (173). TFAM overexpression upregulated mtDNA and total TFAM levels, prevented the reduction of mtDNA copy number and inhibited motor and sensory nerve conduction abnormalities in diseased mice (174). A study on the neurological evaluation of 125 Italian T2DM patients noted that mtDNA was reduced in T2DM patients, this result was more significant in DPN patients and was associated with the MIR499A gene polymorphism (175).

### 4.3 Inactivation and recovery of antioxidant defense systems in diabetic microvascular complications

#### 4.3.1 DKD

CD38 inhibitor apigenin upregulated NAD/NADH ratio and SIRT3-mediated mitochondrial antioxidant enzyme activity, while knockdown of CD38 inhibited SIRT3 activity, suggesting a correlation between CD38 and SIRT3 in oxidative stress mechanisms (176). *In vitro* experiments using high glucose-induced glomerular mesangial cells revealed that high glucose stimulated ROS production, decreased SOD and GSH levels, increased NADPH oxidase activity and promoted an increase in apoptotic factors which was also verified in diabetic rats (177). Antioxidant peptide SS31 inhibited the reduction of MnSOD and CAT activity, inhibited NADPH oxidase and NF-κB p65 activity in db/db mice and high glucose induced HK-2 cells (178). It had also been shown that exercise increased the expression of SOD and reduced oxidative damage (179). In addition, the activity of antioxidant enzymes in the body changed with the duration of diabetic hyperglycemia. The mRNA expression and activity of heme oxygenase-1 (HO-1) and MnSOD increased, and GSH-Px activity increased during short-term hyperglycemia; as the disease progressed the mRNA expression and activity of both decreased, accompanied by an increase in MDA and a decrease in GSH levels (180). The use of fluorofenidone in db/db mice showed that fluorofenidone alleviated oxidative stress-induced renal injury by blocking RAGE/AGEs/NOX and PKC/NOX signaling, down-regulating NADPH oxidase and up-regulating GSH-Px and SOD (181). In another study using STZ to create a model of DM in rats, MDA, CAT and GSH-Px were significantly different compared with the control group and tempol treatment restored GSH-Px levels (182). Intervention with carnosine in H_2_O_2_-induced HK-2 cells concluded that carnosine increased total SOD activity, decreased NOX4 expression and ROS levels, and alleviated oxidative stress (183). The use of honokiol in BTBR ob/ob mice with T2DM resulted in the conclusion that honokiol ameliorated renal damage and maintained mitochondrial function by activating SIRT3 and thereby restoring SOD2 and PGC-1α expression (184). In STZ-induced diabetic rats, Rap1 significantly ameliorated mitochondrial dysfunction and oxidative stress injury in renal tubular cells, modulated C/EBP-β binding to the endogenous PGC-1α promoter, and the interaction of PGC-1α with CAT or SOD (185).

#### 4.3.2 DR

Exendin-4 (a glucagon-like protein) increased GSH and magnesium superoxide dismutase levels, decreased NADPH oxidase levels, inhibited ROS production and cyt-c release, and prevented apoptosis in high glucose-induced adult human retinal pigment epithelial-19 cells by inhibiting p66Shc expression and activation (186). In a study on the relationship between retinal neuronal apoptosis and MnSOD in diabetic rats, it was noted that apoptosis increased in diabetic rats at 8 and 12 weeks, and the number of RGC cells decreased at 12 weeks, while MnSOD activity and mRNA levels decreased at 4, 8 and 12 weeks, indicating a close relationship between MnSOD and RGC apoptosis (187). Similarly, two other studies had shown that MnSOD overexpression inhibited the increase in 8-OHdG and nitrotyrosine levels, prevented the decrease in GSH and total antioxidant capacity caused by DR (188, 189). An interesting study explored the response of knockdown of the Sigma 1 receptor (σ1RKO) on primary retinal Müller glial cells, showing that SOD1, CAT and GPX1 expression and protein levels were reduced in the σ1RKO group, as well as GSH and GSH/GSSG ratios, demonstrating that the neuroprotective effects of σ1R are related to the inhibition of oxidative stress (190).

#### 4.3.3 DPN

Aldose reductase inhibitors corrected neurological and metabolic abnormalities, restored GSH and ascorbic acid levels, and inhibited lipid peroxidation in diabetic rats (191). Berberine (BBR) increased Nrf-2-mediated antioxidant defense system, ameliorated mitochondrial damage and neurotransmission abnormalities in diabetic rats, and upregulated PGC-1α-mediated mitochondrial biogenesis in high glucose-induced N2A cells, demonstrating the important role of BBR in DPN treatment (192).

## 5 Abnormalities in metabolic pathways of oxidative stress

**Figure 4:**
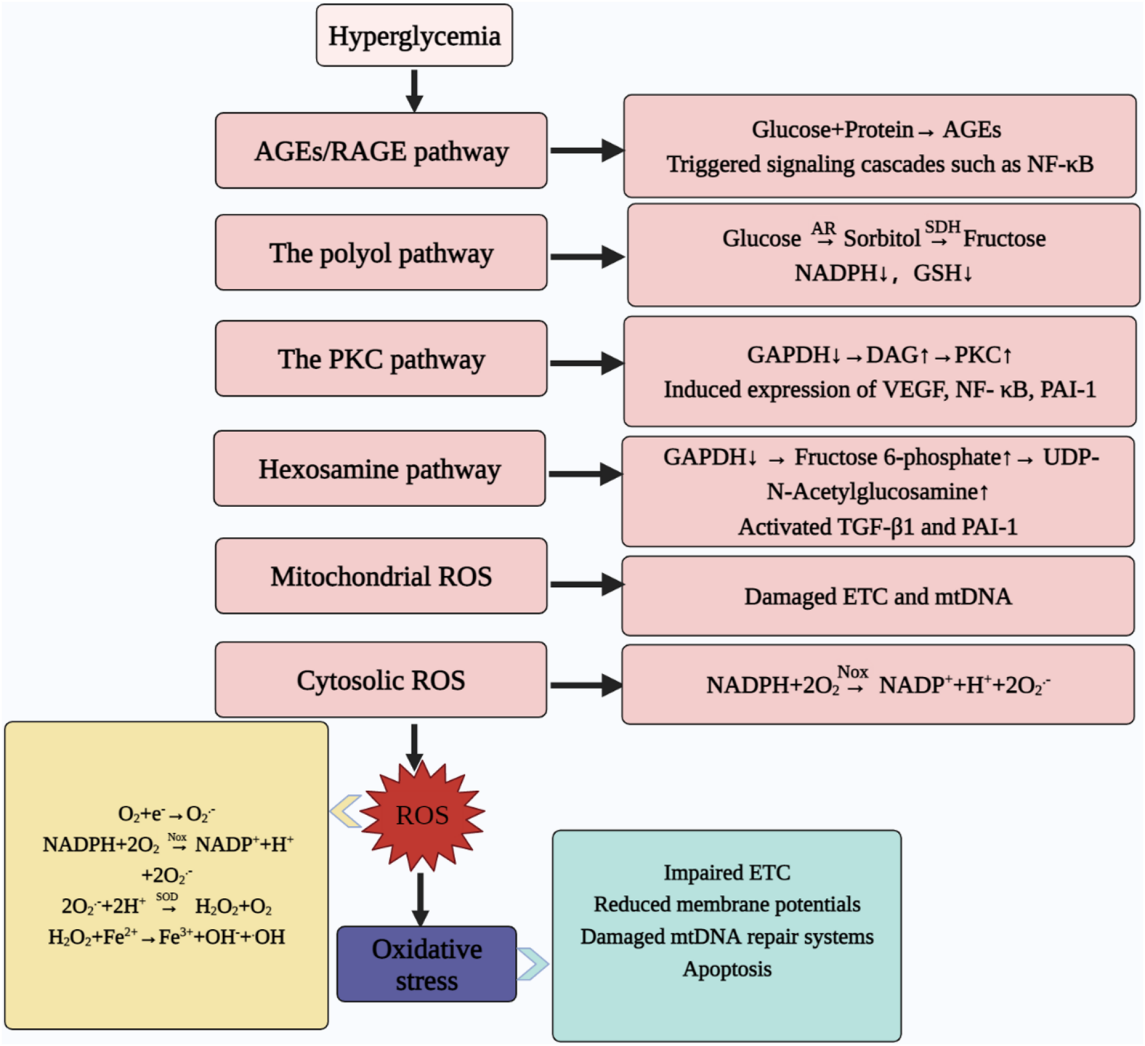
Abnormal metabolic pathways caused by hyperglycaemia. Hyperglycemia contributes to ROS production through the AGEs pathway, hexosamine pathway, PKC pathway, and polyol pathway; meanwhile, mitochondria and cytoplasm are also important sites for ROS production, which ultimately leads to oxidative stress. At the same time, oxidative stress can contribute to ETC abnormalities, reduce MMP, damage the mtDNA repair system and promote apoptosis. AGEs, advanced glycosylation end products; RAGE, the receptor for AGEs; NF-ΚB, nuclear factor kappa-light-chain-enhancer of activated B cells; AR, aldose reductase; SDH, sorbitol dehydrogenase; NADPH, nicotinamide adenine dinucleotide phosphate; GSH, reduced glutathione; GAPDH, glyceraldehyde-3-phosphate dehydrogenase; DAG, diacylglycerol; PKC, protein kinase C; VEGF, vascular endothelial growth factor; PAI-1, plasminogen activator inhibitor-1; TGF-Β1, transforming growth factor; ROS, reactive oxygen species; ETC, electron transfer chain; mtDNA, mitochondrial DNA; NOX, NADPH oxidases; NADP^+^, nicotinamide adenine dinucleotide phosphate oxidized; O_2_^.-^, superoxide anion; SOD, superoxide dismutase; H_2_O_2_, hydrogen peroxide; •OH: hydroxyl radical.

High glucose-induced activation of the AGE, PKC, polyol and hexosamine pathways, as well as the formation of ROS in the mitochondria and cytoplasm, contribute to increased ROS production, and promote mitochondrial dysfunction and induce oxidative stress, mediating cellular dysfunction and accelerating the disease process (32, 193–197) (**Figure 4**). We have previously addressed the formation of ROS in the cytoplasm and mitochondria, so the following section focuses on the other four metabolic pathways.

### 5.1 AGEs/RAGE pathway

Non-enzymatic glycosylation of proteins and other macromolecules caused by prolonged high glucose levels, resulting in a series of dehydration and fracture reactions leading to the production of AGEs, resulting in abnormal protein structure and function, and consequently abnormal physiological function (198). AGEs promote oxidative stress by impairing the ETC to promote ROS formation (199). At the same time, the production of ROS can in turn stimulate the production of AGEs, thus creating a vicious circle (200). In addition, AGEs mediate the activation of downstream inflammatory and fibrotic signaling pathways by binding to cell surface receptors (RAGE) (201–203).

### 5.2 The polyol pathway

High glucose environment promoted activation of the polyol pathway (204). Glucose is converted to sorbitol by aldose reductase (AR) and subsequently oxidised to fructose by sorbitol dehydrogenase (SDH), during which NADPH is consumed as an electron donor (205). However, the reduction of the antioxidant GSH is dependent on NADPH, and the high glucose state accelerates the depletion of NADPH and reduces the antioxidant capacity of the body (205). At the same time, sorbitol can increase the osmotic pressure of cells, or act as a precursor substance for the formation of AGEs to promote the body’s sensitivity to oxidative stress, leading to DPN or DR (23, 206, 207). In addition to this, some researchers verified the relationship between AR and NLRP3 inflammasome: AR inhibitors inhibited the activation of NLRP3 inflammasome, reduced the production of inflammatory factors and mitigated the production of ROS during oxidative stress. It proved that AR participated in the innate immune response induced by NLRP3 inflammasome (208, 209).

### 5.3 The PKC pathway

High glucose promotes increased glycolysis, leading to greater diacylglycerol (DAG) production, while inhibition of the glycolytic enzyme glyceraldehyde-3-phosphate dehydrogenase (GAPDH) increases DAG activity and activates the PKC pathway (210). Activation of the PKC pathway is often accompanied by increased production of inflammatory factors and vascular endothelial growth factor (VEGF), and is closely associated with the development of diabetic complications (193, 211–215).

### 5.4 The hexosamine pathway

The hexosamine pathway is one of the pathways that promote the development of DM and its complications (195). Similarly, high glucose acts as a trigger switch for ROS production, resulting in the inhibition of GAPDH activity and the conversion of increased fructose-6-phosphate to the end product diphosphate uracil-N-acetylglucosamine (UDP-GlcNAc) (216). This is accompanied by an increase in ROS and fibrogenic factors downstream of the pathway, causing oxidative stress in mitochondria and is closely associated with thickening of the basement membrane of DKD (23, 217, 218).

## 6 Discussion

Oxidative stress is an imbalance in the redox state of the body, where excessive production of free radicals or damage to the antioxidant system, leads to a pathological outcome that is closely linked to the development of diseases such as cancer and metabolic disorders (219). ROS is a major component of free radicals, mainly produced in small amounts during OXPHOS in mitochondria, and plays an important role in cell signaling, cell proliferation and antibacterial immunity (220, 221).

However, the prolonged and persistent hyperglycaemic state of DM leads to an increase in cellular respiratory substrates entering the mitochondria, with excess electron donors impairing ETC, contributing to ROS production, mediating the breakdown of the proton electrochemical gradient, impaired MMP, increased cyt-c leakage and inadequate ATP synthesis (56). Due to the lack of histone protection of mtDNA, the high mutability of the non-coding region and the restriction of its loop structure, ROS can further damage mtDNA, leading to a reduction in the copy number of mtDNA, damage the genes responsible for encoding some mitochondrial proteins and impair the function of mitochondria (87, 222, 223). At the same time, the increased ROS can damage the repair system of mtDNA, further deepening the damage to mtDNA and causing functional impairment of mitochondria (224, 225). In addition, the instability of ROS encourages cross-linking with macromolecular proteins, DNA and lipids, altering the structure and function of macromolecules and having toxic effects, further affecting cell function (226). However, ROS can also mediate the activation of downstream signaling pathways such as inflammation and fibrosis, leading to the progression of diabetic microvascular complications such as DKD and DPN (227–229).

Studies have shown that some herbal active ingredients (puerarin, polydatin, quercetin, etc.), vitamin C, vitamin E, α-lipoic acid are important antioxidant strategies (160, 163, 164, 230–233). Targeting mitochondria to overexpress catalase in mice extends lifespan and alleviates oxidative stress in diseases such as metabolic syndrome (234). A clinical trial of the drug elamipretide (a mitochondrial tetrapeptide that interacts with cardiolipin) showed that elamipretide significantly improved clinical symptoms and skeletal muscle performance in Barth syndrome (235). In addition, animal models have demonstrated that enzymatic antioxidants mimics (SOD mimics, GPX mimics and CAT mimics) can scavenge superoxide and inhibit oxidative stress (236–238). Recent studies have shown that bioadhesive hydrogel can promote oral wound healing in DM rats; novel nanoparticle can accelerate wound healing in DM and is an emerging and effective treatment strategy (239, 240). Some combinations of antioxidants have also been shown to have antioxidant effects (241–243). The relevant literatures state that the combinations of ferulic acid and metformin have been shown to improve DM (244). And the combinations of superoxide dismutase, α-lipoic acid, acetyl-L-carnitine, and vitamin B_12_ have been shown to improve sural nerve conduction velocity, amplitude and pain in patients with DPN (233). Therefore, antioxidants play a positive therapeutic role in the treatment of DM.

However, many antioxidants suffer from poor solubility, unstable storage and gastrointestinal degradation, thus limiting the use of oxidants in clinical practice (245). In recent years antioxidant drugs have mainly focused on animal studies and have not been adequately tested in clinical trials, therefore, poorly supported by clinical data. The few drugs that have been clinically studied have not yielded satisfactory results either, and achieving effective drug concentrations in the body is an important issue for modern science. In addition to this, mtDNA, a key structure involved in oxidative stress, is under-researched for drugs targeting mtDNA, leading to a lack of development of antioxidant drugs. We hope to be able to characterize mitochondrial dysfunction in the high glucose state and look forward to providing a bit of new ideas for future experimental studies.

## 7 Conclusion

Hyperglycemia causes redox imbalance and massive production of ROS leading to impairment of OXPHOS, mtDNA function, mitochondrial dysfunction and oxidation of macromolecules and in turn accelerates apoptosis and disease progression. Therefore, oxidative stress is an important mechanism that promotes the development of DM and its complications. Targeted development of antioxidants and the combination of multiple acting antioxidant components may be a strategy for the treatment of DM.

## Author contributions

ZZ is responsible for writing and the conception of the article, QH is responsible for drawing and document sorting, and DZ is responsible for sorting out the ideas of the article. FL, XL, and WQ are responsible for controlling the overall quality of the article and revising the article. All authors contributed to the article and approved the submitted version.
